# Members of the high mobility group B protein family are dynamically expressed in embryonic neural stem cells

**DOI:** 10.1186/1477-5956-11-18

**Published:** 2013-04-27

**Authors:** Ariel B Abraham, Robert Bronstein, Emily I Chen, Antonius Koller, Lorenza Ronfani, Mirjana Maletic-Savatic, Stella E Tsirka

**Affiliations:** 1 Program in Molecular and Cellular Pharmacology, Stony Brook University, Stony Brook, USA; 2 Program in Neuroscience, Stony Brook University, Stony Brook, USA; 3 Stony Brook University Proteomics Center, School of Medicine, Stony Brook, USA; 4 Core Facility for Conditional Mutagenesis, San Raffaele Scientific Institute, Milan, 20132, Italy; 5 Department of Pediatrics, Section on Child Neurology, Jan and Dan Duncan Neurological Research Institute, Texas Children’s Hospital, Baylor College of Medicine, Houston, TX, 77030, USA; 6 The Medical Scientist Training Program, Stony Brook University, Stony Brook, USA; 7 Department of Pharmacological Sciences, Stony Brook University, BST8-192, Stony Brook, NY 11794-8651, USA

**Keywords:** HMGB, Mice, Shotgun proteomics, Subventricular zone

## Abstract

Neural Stem Cells (NSCs) are a distinct group of cells present in the embryonic and adult mammalian central nervous system (CNS) that are able to differentiate into neurons, astrocytes and oligodendrocytes. As NSC proliferation declines with age, factors that regulate this process need to be defined. To search for NSC regulatory factors, we performed a quantitative shotgun proteomics study that revealed that members of the High Mobility Group B (HMGB) family are highly expressed in NSCs. Using a neurosphere assay, we report the differential expression of HMGB 1, 2, 3, and 4 mRNAs in proliferating NSCs isolated from various time points during embryonic development, as well as the dynamic expression of HMGB1 and B2 mRNAs and proteins in differentiating embryonic NSCs. Expression of HMGB2 underwent the most dramatic changes during the developmental ages examined; as a result, we assessed its role in NSC proliferation and differentiation. We report the predominance of small diameter HMGB2^-/-^ neurospheres in comparison to wild-type, which correlated with increased proliferation in these smaller HMGB2^-/-^ neurospheres. Our data suggest that HMGB2 plays a regulatory role in NSC cell proliferation and maintenance pathways.

## Introduction

During embryonic development, neuroepithelial cells in the cerebral cortex display characteristics of radial glial cells [1]. These radial glial cells undergo asymmetric divisions and generate neurons and oligodendrocytes. As development progresses and the cortical mantle becomes thicker, the radial glial cells also express astrocytic markers and intermediate filament proteins (nestin, vimentin, and glial fibrillary acidic protein-GFAP). Some of the radial glial cells remain as NSCs during early postnatal development, finally giving rise to the B cells which function as NSCs in the adult mammalian brain [1]. Neural Stem Cells (NSCs) give rise to progenitor cells that differentiate into the three principal cell types of the CNS: neurons, astrocytes and oligodendrocytes [2,3].

Studies of these putative embryonic and adult NSCs have defined some of the mitogens that regulate NSC proliferation [4-8], but the precise molecular mechanisms underlying stem cell maintenance and self-renewal remain incompletely understood. As the animals age, the total number of NSCs and proliferating neural stem/progenitor cells (NSPCs) declines [9]. A contributor to the decrease in NSC cell numbers in the subventricular zone (SVZ) is the progressive increase in p16^*Ink4a*^ expression [10]. To gain insight into the molecular mechanisms regulating NSC proliferation, studies have focused on regulators of p16^*Ink4a*^ expression, such as high mobility group protein A2 (HMGA2) [11], which is a member of the HMG superfamily of non-histone proteins found in the nuclei of mammalian cells that bind to nucleosomes and the minor groove of DNA in a sequence-independent manner [12-14]. HMGA2 expression is spatially and temporally specified in CNS tissue; it remains high in the embryonic telencephalon (E11-E14.5) and the lateral ventricle of the CNS during the early post-natal period (P0), but decreases throughout post-natal development and adulthood, becoming undetectable in old age (P600) [11]. The microRNA Let7b, which negatively regulates HMGA2 expression [15], is temporally regulated in NSCs [11]. The Let7b-HMGA2 axis regulates p16^*Ink4a*^ expression, which in turn controls NSC proliferation and self-renewal [16].

The HMG superfamily is composed of three subfamilies: HMG-A, HMG-B, and HMG-N [12,17,18], which act as modulators of transcription, replication, recombination, and DNA repair. Following up on the role of HMGA2 in regulating p16^*Ink4a*^ expression and NSC proliferation, additional studies have reported that the HMGA family of proteins maintain NSC/NPC chromatin in the open state during early development [19]. Moreover, HMGA proteins were identified by iTRAQ proteomic analysis as being expressed during oligodendrocyte precursor cell (OPC) differentiation [20], and transcriptional profiling studies have found that NSCs express HMGB1, HMGB2 and HMGB3 mRNA [21]. A recent study in zebrafish reported that HMGB1 is critical for early brain development, since morpholino-knockdown of HMGB1 expression created forebrain defects and ablation of the catecholamine network [22]. HMGB2 is expressed 17-fold higher in the SVZ stem cell niche than in the olfactory bulb (OB) [23], suggesting a specific role for HMGB2 in SVZ NSC proliferation.

We employed here a shotgun proteomic analysis to identify factors involved in NSC proliferation; the analysis uncovered that members of the HMGB family are variably expressed in mouse NSCs. The proteomics findings were confirmed by real-time PCR and immunoblot analyses during NSC proliferation and differentiation. Our data specifically identify the HMGB1 and B2 proteins as factors expressed and modulated in proliferating and differentiating NSCs. We also report that HMGB2 can regulate NSC proliferation and maintenance.

## Materials and methods

### Animals

Experiments were performed within the Stony Brook University (SBU) guidelines regarding the ethical use of animals and were approved by the SBU Institutional Animal Care and Use Committee. The mouse strains used were C57BL/6 (wild-type, wt), HMGB2^-/-^ in the C57BL/6 background and nestin-GFP in the C57BL/6 background.

### Neural stem cell (NSC) isolation, growth, and differentiation

Pregnant mice were euthanized under deep anesthesia at E12.5. Embryos were placed in ice-cold NSC proliferation medium composed of neurobasal medium containing Neurocult proliferation supplement (Stem Cell Technologies) and antibiotic/antimycotic (Gibco). Embryonic mouse brains were dissected and dissociated by trituration. Cells were centrifuged at 800 rpm for 5 minutes, supernatant removed and the pellet resuspended in 10 mL of NSC proliferation media. Viable cells were counted by trypan blue staining. Primary neurospheres were grown in a 5% CO_2_ chamber at 37°C by seeding 8×10^6^ viable cells at a cell concentration of 2×10^5^/mL in NSC proliferation media containing 20 ng/mL of recombinant human epidermal growth factor (rhEGF, Sigma).

Primary neurospheres were passaged after 7 days *in vitro* using the Neurocult Chemical Dissociation Kit (Stem Cell Technologies) according to manufacturer’s protocol. Dissociated NSCs were passed through a 40 μm filter (BD Falcon) to remove debris and trypan blue used to determine the number of viable NSCs. 2×10^6^ NSCs were re-plated in vented T-75 flasks (BD Falcon) at a cell concentration of 5×10^4^ cells/mL in NSC proliferation media containing 20 ng/mL rhEGF. NSCs from each passage were re-plated at the same density (5×10^4^ cells/mL) and rhEGF (20 ng/mL) concentration in NSC proliferation media.

All NSC differentiation assays were performed on early passage NSCs (1 and 2). Neurospheres in passages 1 and 2 were dissociated as above. Glass coverslips were coated with 100 μg/mL poly-D-Lysine (Sigma) and 20 μg/mL Laminin (Sigma) in PBS for 3-4 hours at 37°C. After laminin coating, dissociated NSCs were plated at 5×10^5^ cells/well in 12-well plates in Neurocult differentiation media (Stem Cell Technologies) without rhEGF supplementation. Differentiation media changes were done once per day per well until the NSCs were lysed for collection of RNA or protein. For evaluation of differentiation by immunofluorescence, NSCs were fixed in 4% PFA, washed in PBS, blocked in 5% goat serum and stained overnight at 4 degrees in 0.3% BSA/0.2%TritonX/PBS with mouse anti-βIII tubulin (MAB1637, Millipore, 1:500) to identify neurons, with mouse anti-GFAP (Sigma, 1:1,000) to identify astrocytes, and anti-CNP (Sigma C5922 1:100) to identify oligodendrocytes. Species-specific secondary antibodies (Invitrogen, 1:10,000) and DAPI (Invitrogen, 1:2,000) in 0.3% BSA/0.2% TritonX/ PBS were used for secondary staining. Confocal imaging of differentiated NSCs was performed using a Zeiss 510 Meta confocal microscope.

To assess NSC proliferation, neurosphere cultures were pulse-labeled with bromodeoxyuridine (BrdU, 0.5 uM), which labels cells during S-phase. The spheres were transferred to PDL-coverslips, fixed and processed for BrdU (rat anti-BrdU antibody, Serotec, 1:300) immunofluorescence. To quantify cell death in the neurospheres, the cultures were fixed and labeled with anti-activated caspase 3 (Sigma C8487, 1:500). The cells were counterstained with Hoechst 33342 or DAPI to label cell nuclei. The percentages of BrdU- or caspase 3-positive cells were counted.

### Shotgun proteomic analysis

#### NSC Lysis

NSC lysis buffer was made using 8 M urea (Sigma) in 50 mM NH_4_HCO_3_ (pH7.5) (Sigma) using HPLC-grade water (Thermo Scientific). 1 mg of RapiGest SF (Waters) was reconstituted with 50 mM NH_4_HCO3 to make 2% RapiGest (w/v). 5× Invitrosol LC/MS protein solubilizer was obtained from Invitrogen. Complete Mini, EDTA-free protease inhibitor cocktail was purchased from Roche. To make mass-spectrometry compatible NSC lysis buffer, protease inhibitor (1×), Invitrosol (1×), urea (4 M final) and RapiGest^SF^ (0.1% w/v final) were added to NH_4_HCO_3_ (50 mM final). NSCs were lysed with 100 μL of lysis buffer on ice and the DNA sheared with a 25-gauge needle attached to a 1 mL syringe (BD). A different needle and syringe were used for each biological NSC sample and all technical replicates. Lysates were incubated in a foam pad attached to a vortex for 30 min at 4° to facilitate the solubilization of proteins and centrifuged at 13,200 rpm at 4° for 30 minutes, and the supernatants transferred to new, ice-cooled, non-stick microcentrifuge tubes and left on ice. Insoluble proteins remaining in the NSC debris pellet were solubilized by adding 100 μL of NSC lysis buffer containing 6 M urea (final concentration). Tubes containing insoluble protein pellets and additional lysis buffer were vortexed at 4° for 30 minutes, centrifuged at 13,200 rpm at 4° for 30 minutes, and the supernatants pooled with their respective soluble protein supernatants on ice. For fractionated NSC lysates, NSCs were lysed and fractionated according to manufacturer protocol using the ProteoExtract native membrane protein extraction kit (Calbiochem). Protein determination was preformed using the EZQ Protein Quantitation Kit (Invitrogen).

#### Digestion and preparation of *whole* NSC and *soluble fraction* NSC protein lysates

10 μg protein from each sample was precipitated using methanol-chloroform precipitation. Protein pellets were resuspended in 1× Invitrosol, heated to 60° for 5 minutes, cooled to room temperature, dissolved in acetonitrile (Sigma) and sonicated for 2 hours in a 37° water bath. Proteins were digested with Trypsin (Sigma, 1:100) at 37° overnight, and quenched with 90% Formic acid (10% final) the following day. Peptide pellets were dried down by speed vacuum to almost dry and resuspended in buffer A (5% acetonitrile/95% water/0.1% Formic Acid).

#### Digestion and preparation of *membrane* NSC protein lysates

10 μg of membrane protein was methanol-chloroform precipitated, resuspended in Rapigest, reduced using TCEP (2-Carboxylethyl-Phosphine), alkylated with iodoacetamide (IAM), and digested with trypsin (1:50) overnight. RapiGest was hydrolyzed by adding 90% Formic Acid (10% final) and incubated in a shaking 37° water bath for 4 hours. Samples were dried down by speed vacuum and resuspended in buffer A.

LC Mass Spectrometry analysis**:** NSC peptides were analyzed using a LTQ XL linear ion trap mass spectrometer (Finnigan, Thermo Scientific). MS analysis spectra were extracted from the RAW file with ReAdW.exe (http://sourceforge.net/projects/sashimi). The resulting mzXML file contains the data for all MS/MS spectra and can be read by the subsequent analysis software (Additional file 1). The MS analysis data was searched with Inspect [24] against a database containing a mouse database (IPI ver. 3.43 containing 54215 entries) with added *E.coli* and common contaminant proteins (in total 4605 proteins) in addition to a shuffled database of the aforementioned proteins. Only peptides with at least a p value of 0.01 were analyzed further.

#### qRT-PCR and immunoblots

Mouse NSCs were isolated from the forebrain of NestinGFP mice. Samples were cut in the ventral-dorsal plane immediately caudal to the telencephalon to ensure the separation of the telencephalon from the developing mid/hindbrain and spinal cord. The developmental characteristics of each embryo were verified using the Theiler Atlas of Mouse Development [25].

Total RNA was isolated from proliferating and differentiating NSCs using the RNeasy RNA Isolation Kit (Qiagen) and levels were quantified. Prior to reverse transcription, RNA samples were treated with DNAse (Invitrogen). 750 ng of total RNA (DNAse-treated) was reverse transcribed using the Superscript III First-Strand Synthesis System for RT-PCR (Invitrogen). NSC cDNA was used to conduct quantitative real time RT-PCR (qRT-PCR) using the SYBRGreen PCR kit (Qiagen) with gene specific primers for HMGB1, B2, B3, and B4. Reactions were read in a 7300 Real Time PCR System (Applied Biosystems). β-actin primers served as a normalization control. Primer Sequences were:

HMGB1(L) 5^′^ACAGAGCGGAGAGAGTGAGG 3^′^ and HMGB1(R) 5^′^TTTGCCTCTCGGCTTTTTAG 3^′^;

HMGB2(L) 5^′^ TGTCCTCGTACGCCTTCTTC 3^′^ and HMGB2(R) 5^′^ CCTCCTCATCTTCTGGTTCG 3^′^;

HMGB3(L) 5^′^GCGAACAATACAGGTACGACTC 3^′^ and HMGB3(R) 5^′^ CTTGGCACCATCAAACTTCC 3^′^;

HMGB4(L) 5^′^ CGGGACCACTATGCTATGCT 3^′^ and HMGB4(R) 5^′^ CTTCCTGCCTTGACATTGG 3^′^.

Cycling conditions for HMGB1 were: 95° for 15 min, 94° for 15 sec (melting), 53.2° for 30 sec (annealing), 72° for 30 sec (extension), repeat for 40 cycles, 4° hold until end. Annealing temperatures were modified to 58.1° for HMGB2 and HMGB3, and to 50.3° for HMGB4. Fold change in gene expression was calculated using the comparative CT method [26]. All qRT-PCR reactions were run in quadruplicate (4 technical replicates per sample). At least three different biological samples of NSCs at each time point in development were used for each experiment (n=3 experiments).

For quantitative westerns, proliferating and differentiating NSCs were lysed and protein determination was done by DC assay (Biorad). Equal amounts of protein were loaded into 12 or 15% Tris Glycine SDS-PAGE, transferred to PVDF, blocked with 4% BSA/PBS, and incubated overnight at 4° with primary antibodies: Rabbit anti-HMGB1 (Abcam, 1:1000), Mouse anti-HMGB2 (Abcam, 1:200), Rabbit anti-HMGB3 (Epitomics, 1:2000), and Rabbit anti-HMGB4 (Abcam, 1:250). Mouse anti-α-tubulin (Sigma, 1:2000) was used as a loading control. Membranes were washed in 0.2% PBST and incubated with either Alexa goat anti-Mouse 680 (Invitrogen) and/or IR DYE Donkey anti-Rabbit 800 (Jackson) at 1:10,000. Membranes were analyzed using a LICOR Odyssey Scanner with 700 and 800 nm laser excitation.

SVZ tissue was microdissected from 300 μm-thick coronal brain sections and subjected to protein extraction using lysis buffer (50 mM Tris-HCl, pH7.5, 1 mM EDTA, 1 mM EGTA, 1 mM sodium orthovanadate, 50 mM sodium fluoride, 0.1% 2-mercaptoethanol, 1% triton X-100, plus proteases inhibitor cocktail; SIGMA). Protein samples (10-40 μg) were separated on 10% SDS-PAGE and transferred to PVDF membranes (Millipore, Bedford MA). Primary antibodies from Santa Cruz Biotechnologies (Caramillo CA) were used at 1 μg/ml. Antibodies were used in combination with a secondary horseradish peroxidase-conjugate (Jackson Immunoresearch).

E15.5 embryos were used for immunohistochemical (IHC) analysis of HMGB2 expression. Brains were collected in 4% paraformaldehyde, cryopreserved in 30% sucrose solution overnight, and used to prepare 30 μm sagittal sections which were blocked and then stained with rabbit anti-HMGB2 (Proteintech, 1:250) in 0.3% BSA/0.2% TritonX/PBS solution overnight at 4°C. The sections were washed with PBS and stained with the highly cross-absorbed secondary antibody anti-rabbit rhodamine red X (Jackson 1:500) in 0.3% BSA/0.2% Triton-X/PBS solution at room temperature for 1 hour. Sections were washed extensively with PBS, mounted on Superfrost plus micro slides (VWR), covered with DAPI Fluoromount G mounting media (Southern Biotech) and a coverslip (Fisher). A Zeiss LSM 510 confocal system with an Axiovert 200 M inverted microscope was used to obtain 63× Z-stack images of the entire thickness of sagittal brain sections containing the VZ/SVZ.

#### Statistics

Comparisons were conducted using one-tailed unpaired t tests except for the NSC differentiation qRT-PCR comparisons which were conducted using two-tailed paired t tests. Statistical significance cut-off for all comparisons was p≤0.05.

## Results

### HMG subfamily B (HMGB) mRNAs and proteins in NSCs

**Figure 1:**
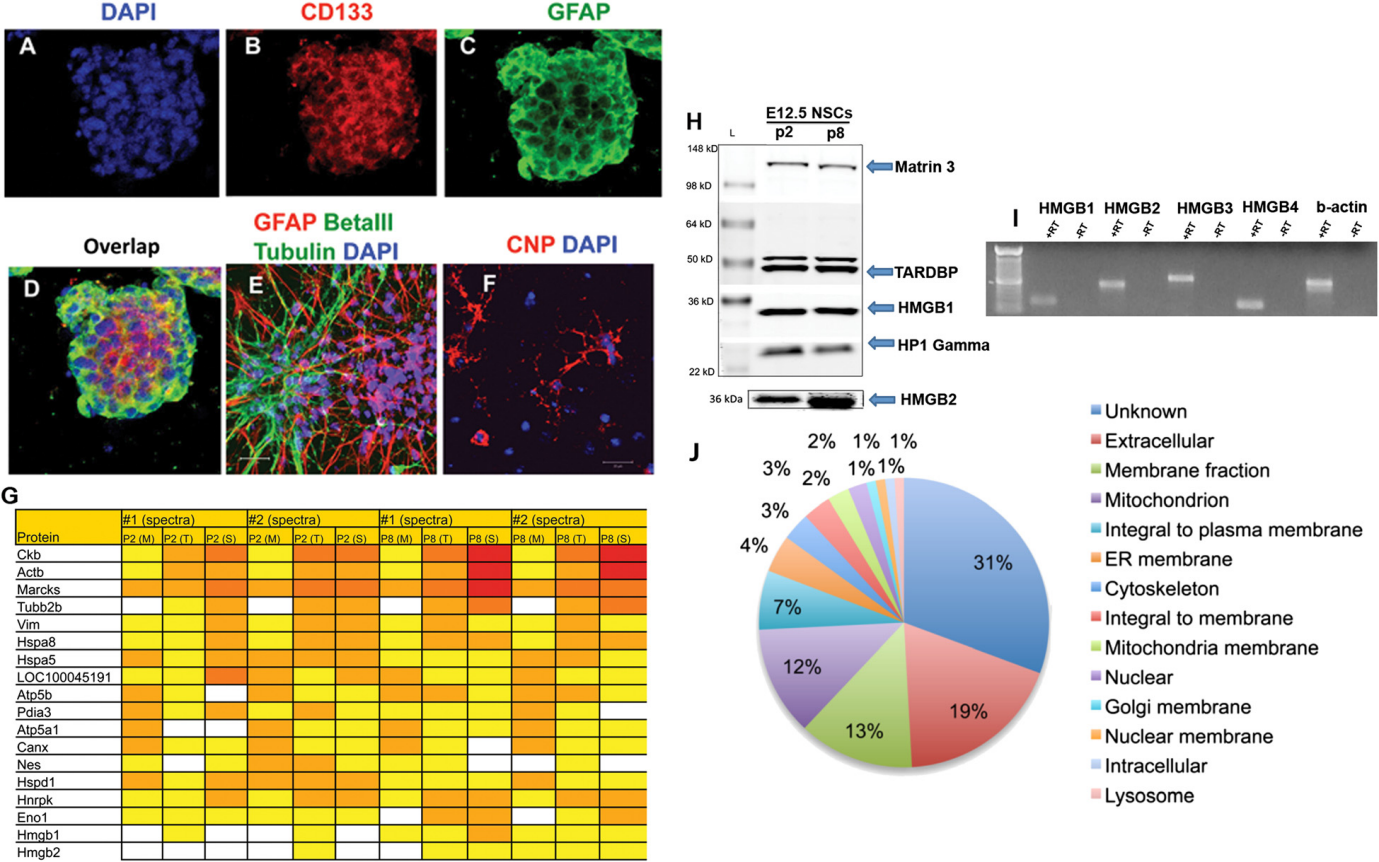
**Shotgun proteomics screen identifies proteins in proliferating E12.5 NSCs including HMGB chromatin proteins. (A-D) **Neurospheres containing proliferating E12.5 NSCs express stem cell markers. **(E-F) **NSCs are multipotent and differentiate into βIII tubulin+ neurons, GFAP+ astrocytes, and CNP+ oligodendrocytes. **(G) **Heat map of protein expression of 20 prominent proteins in proliferating E12.5 NSCs following passage 2 (P2) and passage 8 (P8) with high (red, >30 spectral counts), medium (yellow, 10-30 spectral counts) and low (white, 1-10 counts) levels of expression. #1 and #2 spectra refer to biological replicates, and the heat map is split into membrane fraction (M), total cell lysate (T) and soluble/supernatant (S). **(H) **Immunoblot validation of E12.5 proliferating NSC for proteins identified by the proteomics screen, including Matrin3, TAR DNA binding protein (TARDBP/TDP43), HP1 gamma, and HMGB1 and B2. 30 μg of total protein were loaded on the gel. **(I) **RT-PCR analysis of HMGB1, 2, 3, and 4 mRNA expressions in proliferating E12.5 NSCs. **(J) **Cellular spatial classification of the proteins identified by the screen.

A neurosphere formation assay was used to grow NSCs isolated from the brains of embryonic E12.5 C57BL/6 mice [4-6]. The neurospheres expressed the NSC marker CD133 (Figure 1A-D). They also expressed glial fibrillary acid protein (GFAP) [27,28] and were highly proliferative, capable of extensive self-renewal and multipotent differentiation, generating astrocytes (GFAP+), neurons (βIII tubulin+) and oligodendrocytes (CNP+) when plated on laminin and poly-D-lysine (Figure 1E,F). We subjected the neurospheres after their 2^nd^ (P2) and 8^th^ (P8) passage to shotgun proteomics [29]. Using the global profiling approach, we identified 383 proteins expressed in soluble, membrane, and whole cell lysates (Additional file 1: Figure S1). The analyses identified several NSC protein markers including GFAP, nestin, vimentin, and brain lipid binding protein (BLBP), as well as several hundred proteins that have not previously been reported in NSCs.

A heat map of the 20 most abundant NSCs proteins identified in the MS analysis are shown in Figure 1G. Nuclear proteins including Matrin3, HMGB1 and HMGB2 and Myristoylated Alanine-Rich C kinase Substrate (MARCKS) were detected. MARCKS is a protein kinase C substrate involved in regulation of actin filament crosslinking [30], which plays a role in embryonic radial glial proliferation and positioning during mouse cortical development [31]. In addition, we identified MARCKS-like protein (MLP), a substrate for protein kinase C; Chromobox1 (Cb×1), the mammalian homolog of *Drosophila* heterochromatin protein HP1-β that regulates NSC proliferation and plays a role in mouse cortical development [32]; and Chromobox3 (Cb×3) (mammalian HP1-γ). Our analysis also revealed numerous arginine-serine-rich RNA splicing factors (Sfrs1, Sfrs3, Sfrs4, Sfrs7, Sfrs9, and Sfrs10) and RNA-binding proteins (Fus and Tardbp), suggesting a prominent role for RNA function and metabolism components in proliferating embryonic radial glia / NSCs.

Several proteins identified by proteomic analysis were chosen for validation and confirmed by western blot analysis including HMGB1 and 2, Hp1 gamma, Matrin3, and TARDBP (Figure 1H). To verify the expression of HMGB1 and HMGB2 in proliferating NSCs and explore whether the remaining HMGB family members, HMGB3 and HMGB4, are also expressed, we used RT-PCR. HMGB1, 2, 3, and 4 were expressed in proliferating E12.5 NSCs (Figure 1I). All primers spanned exon-intron boundaries (except B4 which is an intron-less gene) and the size of all RT-PCR reaction amplification products were consistent with amplicons of the individual HMGB cDNAs. The proteins identified from the proteomics screen were clustered based on their predicted cellular localization (Figure 1J).

### HMGB2 is expressed in proliferating and differentiating NSCs

**Figure 2:**
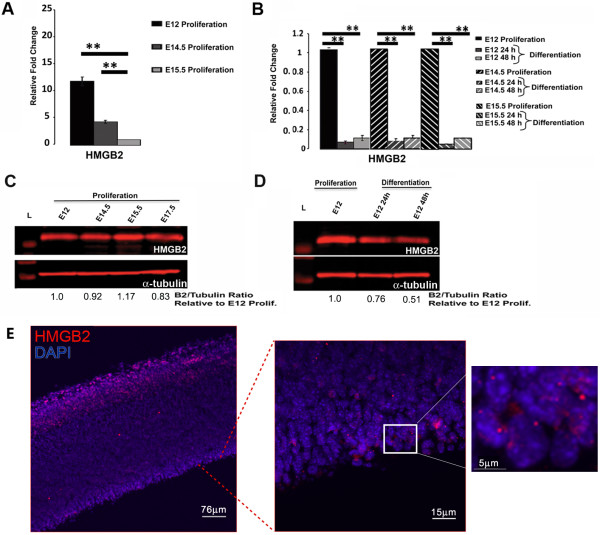
**Proliferating and differentiating NestinGFP+ NSCs isolated from E12-E15.5 embryonic forebrains differentially express HMGB2. (A) **qRT-PCR analysis of HMGB2 mRNA expression in proliferating forebrain NSCs isolated at different time points during neural development. **(B) **qRT-PCR analysis of HMGB2 mRNA expression in differentiating forebrain NSCs isolated at E12, E14.5, and E15.5 and compared to age-matched proliferating NSCs. Note, in differentiating NSC analysis proliferating NSCs were set to 1. **(C) **HMGB2 protein is expressed in proliferating E12-E17.5 NSCs and is differentially expressed in differentiating E12 NSCs. Quantitative western blots of HMGB2 in proliferating NSCs from embryonic day E12 to E17.5. **(D) **Quantitative western blot of HMGB2 in differentiating NSCs at 24 and 48 hours after initiation of differentiation. All values are mean+/- SEM, *p≤0.05, **p≤0.005. **(E)***In vivo* expression pattern of HMGB2 at E15.5 in the VZ/SVZ and cortical plate.

**Figure 3:**
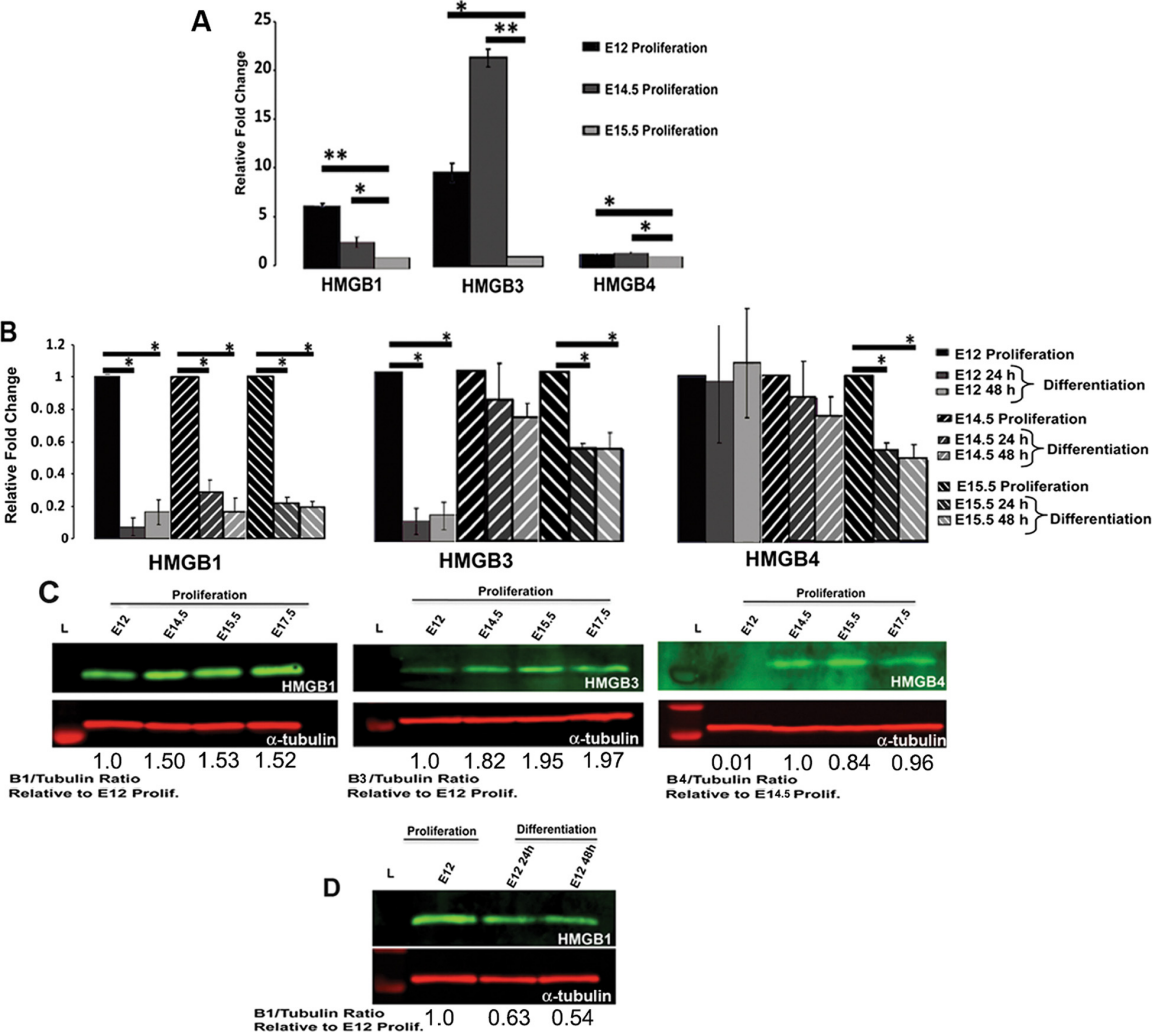
**Proliferating and differentiating NestinGFP+ NSCs isolated from E12-E15.5 embryonic forebrains differentially express HMGB family members. (A) **qRT-PCR analysis of HMGB1, HMGB3, and HMGB4 mRNA expression in proliferating NSCs isolated at different time points during neural development. **(B) **qRT-PCR analysis of HMGB1, HMGB3, and HMGB4 mRNA expression in differentiating forebrain NSCs isolated at E12, E14.5, and E15.5 and compared to age-matched proliferating NSCs. Note, in differentiating NSC analysis proliferating NSCs were set to 1. **(C) **HMGB1, 3, and 4 proteins are expressed in proliferating E12-E17.5 NSCs, and B1 and B2 proteins are differentially expressed in differentiating E12 NSCs. Quantitative immunoblots of HMGB1, HMGB3, and HMGB4 in proliferating NSCs from embryonic day E12 to E17.5. **(D) **Quantitative western blot of HMGB1 in differentiating NSCs at 24 and 48 hours after initiation of differentiation. All error bars are mean+/- SEM, *p≤0.05, **p≤0.005.

To further examine HMGB2 expression, we used NSCs isolated from the forebrain of NestinGFP (Figure 2) transgenic mice [33] at different time points during embryonic neural development. The presence of the transgene allowed for better visualization and accurate dissection of the relevant forebrain structures. qRT-PCR and quantitative western blots were employed to assess changes in HMGB2 mRNA and protein expression in proliferating and differentiating NSCs. We used NestinGFP+ neurospheres isolated between E12 and E17, a highly dynamic time period in neural development that is associated with NSC proliferation and differentiation. NSC proliferation takes place in the medial and lateral ganglionic eminences (MGE and LGE), and NSC differentiation is evident during cortical neurogenesis. HMGB2 mRNA expression was 11.7 fold higher in proliferating NSCs at E12 than at E15.5 (Figure 2A), following a pattern similar to that of HMGA2 mRNA expression between E14.5 and P0 [11]. In differentiating NSCs, changes in HMGB mRNA expression in the differentiating spheres were calculated relative to proliferating NSCs at each developmental time point, revealing that B2 mRNA expression was decreased by approximately 10-fold throughout the period examined (Figure 2B). To assess HMGB protein expression dynamics in proliferating and differentiating NSCs, quantitative western blots were performed. HMGB2 protein expression remained constant in proliferating NSCs isolated from E12 to E17.5 (Figure 2C) and decreased in differentiating NSCs (Figure 2D). To assess corresponding HMGB2 expression *in vivo,* we sectioned and stained E15.5 WT brains for the presence and localization of HMGB2. The protein was found to be concentrated in the ventricular zone (VZ) and SVZ as well as in the marginal zone and the developing cortical plate [34] (Figure 2E). The expression of mRNA and protein for other members of the HMGB family was found to vary in proliferating and differentiating NSCs (Figure 3). HMGB1 mRNA expression was 5.9-fold higher in proliferating NSCs at E12 than at E15.5. HMGB3 mRNA expression was 9.6-fold and 21.3-fold higher in proliferating E12 and E14.5 NSCs. For HMGB4 mRNA expression, the changes were very small in proliferating NSCs, arguing against a role for HMGB4 in regulating NSC proliferation (Figure 3A). In differentiating NSCs, HMGB1 mRNA expression decreased at all time points assessed by approximately 5-fold. HMGB3 mRNA expression decreased 10-fold in differentiating E12 NSCs, but did not change significantly in differentiating E14.5 and E15.5 NSCs. HMGB4 mRNA expression was unchanged in differentiating NSCs at E12 and E14.5, but decreased in differentiating E15.5 NSCs (Figure 3B). Using quantitative immunoblots (Figure 3C), HMGB1 protein expression remained constant in proliferating NSCs isolated from E12.5 to E17.5. Low HMGB3 protein expression was detected in proliferating E12 NSCs, sharply increased at E14.5, and remained high in proliferating NSCs between E14.5 and E17.5. HMGB4 protein expression was not detectable in E12 NSCs, but was present and stable between E14.5-E17.5 in proliferating NSCs. In differentiating NSCs, a decrease in HMGB1 protein levels was evident (Figure 3D). The *in vivo* spatial expression profiles of HMGB1, HMGB3 and HMGB4 have been documented before and are available in the Allen Brain Atlas searchable database (http://developingmouse.brain-map.org/).

### HMGB2^-/-^ SVZ neurospheres isolated from embryonic mice are smaller than HMGB2^+/+^ SVZ neurospheres *in culture*

**Figure 4:**
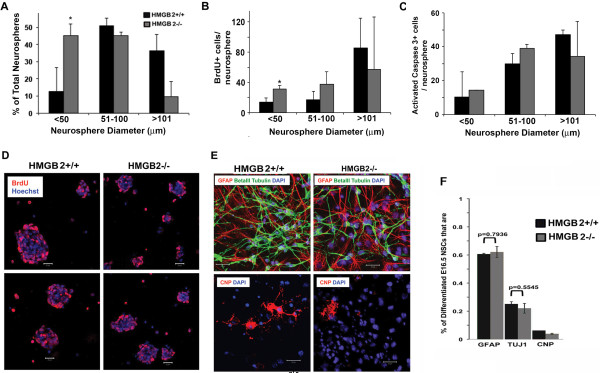
**Embryonic HMGB2**^**-/- **^**neurospheres differentiate similarly to wt ones. **Quantitative analysis of the distribution of HMGB2^-/- ^neurosphere sizes **(A) **in E16.5 HMGB2^-/- ^neurospheres compared to WT E16.5 neurospheres,** (B) **numbers of BrdU+ cells/sphere,** (C) **numbers of activated caspase 3+ cells/sphere**. (D) **BrdU+ labeled spheres from HMGB2^-/- ^and WT E16.5 mice. **(E, F)** Differentiation of E16.5 HMGB2^-/- ^neurospheres give rise to GFAP+, βIII tubulin+, and CNP+ cells. N=4 biological replicates for each genotype and 15 neurospheres/biological replicate. Scale bar: 20 μm. * indicates that p≤0.05.

Our data indicated dynamic expression of HMGBs in embryonic NSCs and the most dramatic changes in HMGB2 during proliferation and differentiation. We thus explored the proliferation and differentiation properties of NSCs in HMGB2^-/-^ mice, which had previously been described to have reduced fertility tied to defects in spermatogenesis [35] as well as perturbations in chondrocyte development [36]. Using the neurosphere system to assess proliferation and differentiation, E16.5 neurospheres were assayed for possible variations in size, number, BrdU incorporation, and capacity for multi-lineage differentiation after plating on laminin- and poly-D-lysine-coated coverslips. Cell marker analysis was also performed. E16.5 HMGB2^-/-^ neurospheres were smaller than WT E16.5 neurospheres (Figure 4A,D). Counterstaining with Hoechst allowed for visualization of the nuclei, revealing that neurospheres of similar sizes contained comparable numbers of cells. When proliferation was assessed using BrdU incorporation (Figure 4B,D), differences were observed only in the smaller neurospheres: higher numbers of BrdU+ cells were observed in the HMGB2^-/-^ neurospheres with diameter less than 50 μm. Apoptotic cell death, as visualized by immunofluorescent staining for activated caspase 3, was similar between the two genotypes in all neurospheres. Moreover, differentiation conditions did not result in altered proportions of astrocyte (GFAP+ cells), neuronal (βIII tubulin+), or oligodendrocyte (CNP+) cell populations (Figure 4E-F).

## Discussion

In an effort to determine markers that affect NSC maintenance, quantitative shotgun proteomic analysis of proliferating E12.5 NSCs was used to identify 383 proteins of which a select number were validated by quantitative immunoblot analysis. We focused on the chromatin structural proteins of the HMGB family and confirmed that HMGB1, 2, 3, and 4 were present in proliferating NSCs both at the mRNA and protein levels. Although HMGB1, 2, and 3 mRNA expression changed over time in proliferating NSCs, the HMGB1, 2, and 3 protein expression was stable (Figures 1, 2, 3). During differentiation HMGBs are expressed differentially in NSCs, with decreased HMGB1 and 2 mRNA and protein expression 24 hours after initiating NSC differentiation.

The HMGB1 and B2 expression patterns we report here parallel the HMGA2 expression pattern in NSCs [11]. HMGA2 expression is higher in the NSC proliferative compartment of the embryonic telencephalon (ventricle zone) than it is in the differentiated compartment (cortex). Both HMGB1 and B2 expression is high at early stages of development (during the proliferative phase) and decreases substantially in differentiating NSCs. These expression patterns suggest possible roles for these proteins in the proliferation of radial glia in the embryonic mouse brain, and potentially during repair mechanisms after injury in the adult. Consistent with this idea, findings from studies on repair mechanisms following intracerebral hemorrhage suggest a role of HMGB1 in neurogenesis [37]. Since HMGB2 underwent a more dramatic change in expression than HMGB1, we focused our attention on it. In agreement with our findings, Taniguchi et al report in human mesenchymal stem cells that HMGB2 expression was high in proliferating cells and decreased as the cells differentiated [38].

An intriguing finding of our data is the decrease in HMGB2 mRNA levels in proliferating NSCs between E12 and E15.5 (Figure 2A) while the protein expression level remained stable (Figure 2C), presumably due to the long half-lives that the HMGB proteins are reported to have (~65 hours). The results suggest that there may be negative regulation of HMGB2 mRNA expression in proliferating NSCs during neural development. MicroRNA(s) have been shown to regulate expression of genes in neural progenitors in the adult SVZ neurogenic cascade [39-41], so it is possible that they modulate HMGB2 expression as well. Using MicroCosym Targets Version 5 and miRBase (Enright Lab, European Bioinformatics Lab), an analytic tool which applies the miRanda algorithm to search for putative microRNA binding sites in target mRNAs [42,43], we found putative miRNA binding sites in the HMGB1 mRNA and 45 different miRNA binding sites in HMGB2. Among them were several binding sites for members of the Let-7 family of microRNAs, including Let-7a,f,g and Let7-b, which have been reported to negatively regulate HMGA2 in NSCs [11]. Several miRNAs have been reported to regulate the expression of members of the HMGB family in different systems [44-46], however it remains unclear what role microRNAs have in the regulation of HMGB1 or B2 expression in the embryonic neurogenic niche.

HMGB2 deficiency resulted in neurospheres of smaller overall size than that observed for wild-type neurospheres (Figure 4), a change that was accompanied by increased proliferation. It is conceivable that the significant increase in small spheres (<50 μm) in absence of HMGB2, along with the increased BrdU incorporation within those clones, is indicative of greater numbers of symmetric divisions [47] of the NSCs. This result suggests that a physiological role for HMGB2 is the possible suppression of proliferation and growth by modifying chromatin structure in NSCs. Consistent with this idea, using a heart failure model that results in heart hypertrophy and increased proliferation of cardiomyocytes, Franklin *et al.* reported that HMGB2 attenuates pathologic cell growth and regulates the expression of genes that are responsible for hypertrophic cell growth. This property was not shared by HMGB1 [48]. It is possible, therefore, that the decrease of HMGB2 expression in the NSCs during embryonic development allows for proliferation and growth of the neural stem cell population.

## Conclusion

In conclusion, we report here that HMGB chromatin structural proteins are differentially expressed in proliferating and differentiating NSCs, suggesting the possibility that HMGBs play a regulatory role in NSC processes. Although HMGB2 knockout mice were previously generated, characterized, and found to exhibit defects in spermatogenesis [35] and chondrocyte development and differentiation [49], neurogenesis in the mice has not been addressed. Currently, we are investigating the role of HMGB2 in proliferation, survival, and differentiation of embryonic and adult NSCs.

## Competing interests

The authors declare that they have no competing interests.

## Authors’ contributions

ABA: Designed and performed experiments, analyzed data, wrote drafts of the manuscript. RB: Designed and performed experiments, analyzed data, wrote drafts of the manuscript. EIC, AK: Designed experiments, analyzed data, wrote drafts of the manuscript. LR: Provided the HMGB2-/- mice. MMS: Designed experiments, analyzed data, edited drafts of the manuscript. SET: Designed experiments, analyzed data, wrote drafts of the manuscript. All authors read and approved the final manuscript.

## Supplementary Material

Additional file 1: Figure S1 List of proteins identified in the proteomic screen. This comprehensive list of proteins obtained from the shotgun proteomics analysis includes accession numbers, IPI protein ID, lists of total spectra, % coverage as well as the total number of peptides identified for each of the 383 proteins uncovered. Click here for file
